# Anserine Reverses Exercise-Induced Oxidative Stress and Preserves Cellular Homeostasis in Healthy Men

**DOI:** 10.3390/nu12041146

**Published:** 2020-04-19

**Authors:** Ahmad Alkhatib, Wen-Hsin Feng, Yi-Jen Huang, Chia-Hua Kuo, Chien-Wen Hou

**Affiliations:** 1 Laboratory of Exercise Biochemistry, University of Taipei, No.101, Sec. 2, Zhongcheng Road., Shilin District, Taipei City 11153, Taiwan; rtp951@yahoo.com.tw (W.-H.F.); kuochiahua@gmail.com (C.-H.K.); 2 School of Health and Life Sciences, Teesside University, Tees Valley, Middlesbrough TS1 3BA, UK; 3 Office of Physical Education, Soochow University, No. 70, Linhsi Road, Shihlin District, Taipei City 11111, Taiwan; yijen@scu.edu.tw

**Keywords:** anserine, nutraceutical, exercise recovery, oxidative stress, cell damage, haematocrit

## Abstract

The study tested whether anserine (beta-alanyl-3-methyl-l-histidine), the active ingredient of chicken essence affects exercise-induced oxidative stress, cell integrity, and haematology biomarkers. In a randomized placebo-controlled repeated-measures design, ten healthy men ingested anserine in either a low dose (ANS-LD) 15 mg·kg^−1^·bw^−1^, high dose (ANS-HD) 30 mg·kg^−1^·bw^−1^, or placebo (PLA), following an exercise challenge (time to exhaustion), on three separate occasions. Anserine supplementation increased superoxide dismutase (SOD) by 50% (*p* < 0.001, effect size d = 0.8 for both ANS-LD and ANS-HD), and preserved catalase (CAT) activity suggesting an improved antioxidant activity. However, both ANS-LD and ANS-HD elevated glutathione disulfide (GSSG), (both *p* < 0.001, main treatment effect), and consequently lowered the glutathione to glutathione disulfide (GSH/GSSG) ratio compared with PLA (*p* < 0.01, main treatment effect), without significant effects on thiobarbituric acid active reactive substances (TBARS). Exercise-induced cell damage biomarkers of glutamic-oxaloacetic transaminase (GOT) and myoglobin were unaffected by anserine. There were slight but significant elevations in glutamate pyruvate transaminase (GPT) and creatine kinase isoenzyme (CKMB), especially in ANS-HD (*p* < 0.05) compared with ANS-LD or PLA. Haematological biomarkers were largely unaffected by anserine, its dose, and without interaction with post exercise time-course. However, compared with ANS-LD and PLA, ANS-HD increased the mean cell volume (MCV), and decreased the mean corpuscular haemoglobin concentration (MCHC) (*p* < 0.001). Anserine preserves cellular homoeostasis through enhanced antioxidant activity and protects cell integrity in healthy men, which is important for chronic disease prevention. However, anserine temporal elevated exercise-induced cell-damage, together with enhanced antioxidant activity and haematological responses suggest an augmented exercise-induced adaptative response and recovery.

## 1. Introduction

The consumption of chicken essence is widely popular across parts of south and east Asia. It has long been associated with alleviating physical and mental fatigue [1]. The two key bioactive ingredients identified within the chicken breast muscle for extraction are anserine (beta-alanyl-3-methyl-l-histidine) and carnosine [1]. Anserine is a methylated analogue of carnosine and possesses a similar chemical structure (C_10_H_14_N_4_O_3_ and C_9_H_14_N_4_O_3_ for anserine and carnosine, respectively). The stabilizing properties of anserine or carnosine on biological membranes are based on the ability of these dipeptides to interact with lipid peroxidation products and reactive oxygen species (ROS) to prevent cellular membrane damage [2].

Previous studies have focused on chicken essence when attributing anserine and carnosine’s various antioxidative, antiglycating, anti-inflammation, antilipogenic, and antifatigue protective properties [3,4,5,6,7]. Others have only focused on carnosine’s antioxidative role [8]. For example, diabetic rats who were administered carnosine (250 mg·kg^−1^·bw^−1^ for four weeks), showed a 24% reduction in serum reactive oxygen species, 20% reduction in advanced glycation end products, and 36% reduction in oxidant-mediated protein damage [8]. However, anserine has shown more bioavailability in humans compared with carnosine, and a higher resistance to hydrolysis by its degrading dipeptide carnosinase [9]. A higher antioxidation for anserine than carnosine and a similar free radical scavenging effect have also been reported following anserine extraction from chicken [3,4].

To date, anserine supplementation effects on oxidative capacity cellular integrity and homeostasis biomarkers in humans are largely unexplored, especially under physiological stress. Oxidative stress is known excess formations of ROS, which alters biological structure by modifying cell membranes, lipids, proteins, and nucleic acid, resulting in cell injury and death. The control of stable ROS in cell compartments and under stress situations, such as post exercise challenge are often indicated by the cellular activities of superoxide dismutase (SOD) and catalase (CAT) enzymes, and nonenzymatically in the cytosol by glutathione (GSH) and glutathione disulfide (GSSG) antioxidant protective mechanisms [2,10]. Whereas, the cell membrane protection against ROS is indicated by lowering the formation of thiobarbituric acid active reactive substances (TBARS) at different tissues, which is found following carnosine intake within chicken extract [10].

Intense bouts of exercise are known to induce changes in the aforementioned oxidative stress biomarkers, in addition to altering cell integrity and homeostasis through depleting skeletal muscle satellite cells [11]. High intensity exercise produces ultrastructural muscular disruption, impaired excitation-contraction coupling, preferential fibre type damage, and impaired muscle metabolism, which in turn causes delayed onset of muscle soreness, swelling of the affected limb, decreased range of motion, and impaired muscle force producing capacity [12]. Alterations in cell damage biomarkers such as glutamic-oxaloacetic transaminase (GOT), glutamate pyruvate transaminase (GPT), creatine kinase isoenzyme (CKMB) and myoglobin, and associated adverse haematological responses of red blood cells (RBCs) and white blood cells (WBCs) have all been previously reported following single or repeated bouts of intense exercise [13,14,15]. For example, CKMB and myoglobin are known muscle damage biomarkers, and their levels increase several-fold immediately after exhaustive endurance exercise and can take up to 48 h to be restored close to its baseline levels, depending on the severity of exercise or the endurance of sporting event [14]. Two-fold increases in blood GOT and GPT have also been reported immediately after intermittent high intensity sports training [13]. Given the promising effects of chicken essence found in restoring physical strength and reducing mental fatigue following intense exercise [6,16], it would be important to investigate the potential restorative mechanisms in which anserine protects against exercise-induced oxidative stress and cell damage, and whether such effects impact exercise performance.

The present study investigates whether and how anserine supplementation affects exercise-induced oxidative stress, cellular damage, and haematology in healthy adults. We hypothesized that anserine restores cellular integrity and protects against oxidative stress following an acute bout of exercise challenge.

## 2. Methods

### 2.1. Study Design and Participants

The study was a randomized crossover placebo-controlled, repeated measures trial. All experimental tests were performed at the same location (Laboratory of Exercise Biochemistry, University of Taipei), at a similar time of the day, and at similar laboratory environmental conditions, which were controlled for air temperature, relative humidity, and barometric pressure. The sample size provided a power of 90% at 5% significance alpha level, based on the least meaningful differences induced by anserine supplementation on oxidative stress biomarkers. Ten participants were recruited, who were all healthy active men volunteers. Participants were all college students, who reported being physically active (trained 2–3 sessions per week, moderate to intense activities such as walking, gym, cycling, basketball) during the previous six months. Their physical characteristics are: (Mean ± standard deviation; age = 20.9 ± 1.7 years, height = 1.73 ± 0.14 m, body weight (bw) = 69.5 ± 2.5 kg, peak oxygen uptake ($\dot{V}$O_2peak_) = 56.4 ± 2.6 mL·kg^−1^·min^−1^). Exclusion criteria consisted of any history of chronic diseases such as cardiovascular, diabetes, musculoskeletal or neuromuscular or neurological disease; medications, nutritional supplement or ergogenic aids, including any that contains anserine. Each participant underwent an interview with a qualified nutritionist, who discussed foods, which may contain anserine to ensure exclusion from the diet for at least two weeks before the study. Participants were asked not to change their dietary habits throughout the study and were advised to refrain from consuming certain anserine-rich foods beyond the usual habitual intake (e.g., avoid chicken extract supplements common in Asia, avoid any excess intake of chicken, fish, or bird-based foods). All participants confirmed no changes to their dietary habits throughout the duration of the study. The study was approved by the Ethical committee of the University of Taipei. All participants gave their written informed consent and the study procedures adhered to the Declaration of Helsinki guidelines.

### 2.2. Procedures and Protocol

**Figure 1:**
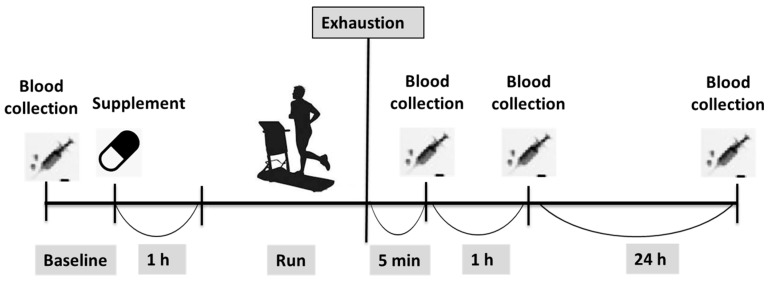
Experimental procedures of the study.

The participants reported to the laboratory, following an overnight fast, on four separate occasions, which were each separated by a two-week interval. The first visit involved a baseline assessment, which involved an incremental exercise running test to determine $\dot{V}$O_2peak_ and anthropometric measurements. The following three visits involved exercise running time trials to volitional exhaustion (TTE) at an individualized speed and gradient calculated based on the corresponding to 50%$\dot{V}$O_2peak_, and were each preceded by anserine supplementation in either a low dose, a high dose, or placebo in random order, and involved blood sampling and biochemistry analyses of oxidative stress, cellular damage, and haematocrit biomarkers (Figure 1).

### 2.3. Baseline Assessment

All participants undertook an incremental running $\dot{V}$O_2peak_ test on a treadmill (Runner SNC, Cavezzo, Italy), with a continuous respiratory gas exchange measurement using a gas analyzer (MetaMax 3B, Cortex Biophysik GmbH, Leipzig, Germany). The Bruce protocol for $\dot{V}$O_2peak_ was used, in which the treadmill speed was initiated at 2.7 km·h^−1^ and increased as follows (4.0, 5.4. 6.7, 8.0, 8.8, 9.6 km·h^−1^), while the gradient was initiated at 10% and increased by 2% every 3 min until volitional exhaustion, as previously described [17]. Reaching $\dot{V}$O_2peak_ was defined based on meeting at least two of the following criteria: Oxygen uptake ($\dot{V}$O_2peak_) reaching a plateau (an increase of ≤ 2 mL·kg^−1^·min^−1^); heart rate (HR) reaching within 10 beats per min of age-predicted maximal heart rate (220 age), and/or respiratory exchange ratio (RER) > 1.10. $\dot{V}$O_2peak_ was then determined from the individual $\dot{V}$O_2peak_ breath by breath data as the average of the last 15 s.

### 2.4. TTE

The average TTE speed was 6.3 ± 0.3 km·h^−1^ and average gradient was 12.8% ± 0.4%, which corresponded to 71.6% ± 2.5% and 64.6% ± 1.2% relative to the participant’s maximum speed and gradient at $\dot{V}$O_2peak_, respectively. Exhaustion following TTE was defined based on the participant’s inability to sustain the run despite strong verbal encouragement, and participants were asked to straddle the belt and hold onto the handrail on completion. Verbal encouragement was similarly provided for all tests (similar tone, duration, and words).

### 2.5. Supplementation Protocol

Each participant ingested either a low dose anserine (ANS-LD) of 15 mg·kg^−1^·bw^−1^, a high dose anserine (ANS-HD) of 30 mg·kg^−1^·bw^−1^, or a placebo (PLA) tablet, with an equivalent dosage amount. The ingestion timing was set at exactly 1 h before performing each TTE. The doses and timing are in line with the anserine expected bioavailability and time-course biosynthesis [9]. Anserine or the placebo tablets were presented to each participant, according to their body weight, in similar colour, size, and appearance. A single anserine tablet contained 150 mg of standardized anserine extract (Stamina sports, Shinjuku, Japan) and a single placebo tablet contained a similar amount of corn starch.

### 2.6. Blood Sampling and Biochemistry

Venous blood samples were drawn following an overnight fast using vacutainers at four time points: (a) At rest, (b) 5 min post exercise, (c) 60 min post exercise, and (d) 24 h post exercise. Plasma samples were separated by centrifugation at 3000 rpm for 10 min and stored at −80 °C for further assays.

Serum plasma GOT, GPT, CKMB, and myoglobin were analyzed by the enzymatic rate method based on their respective reagents using a chemistry analyzer (DxC 800, Beckman Coulter, Chaska, MN, USA). TBARS, SOD, and CAT were analyzed by the enzyme-linked immunosorbent assay kit (Cayman Chemical, Ann Arbor, MI, USA), and GSH and GSSG were analyzed by the Glutathione fluorometric assay kit (BioVision, K264-100, Milpitas, CA, USA), using an ELISA reader (Infinite M200 Pro, Tecan Group Ltd., Mannedorf, Switzerland).

### 2.7. Data Analysis and Statistics

All data were described as means ± SEM. Differences between anserine and placebo supplementation were analyzed using the two-way repeated-measures ANOVA with supplementation as a within factor and post exercise time points as a between factor. The post hoc Bonferroni test was applied to analyze the differences at each time point. All analyses were conducted using SPSS V 21.0 with the significance level set at *p* < 0.05.

## 3. Results

### 3.1. Antioxidatants Response to Anserine Supplementation

**Figure 2:**
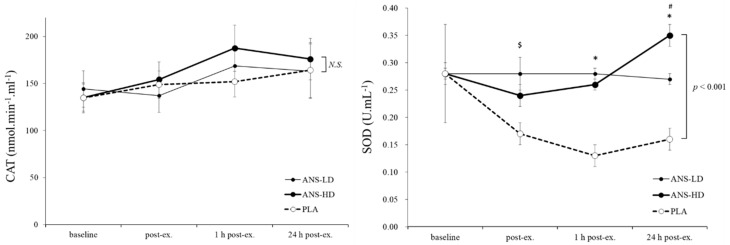
Superoxide dismutase (SOD) and catalase (CAT) responses pre-exercise (baseline), immediately post exercise (post-ex), and following 1 and 24 h recovery with anserine low dose (ANS-LD), high dose (ANS-HD), and placebo (PLA) treatments. Values are mean ± SEM. SOD was significantly increased by both ANS-HD and ANS-LD (*p* < 0.001, ANOVA main treatment effect). * Significantly higher in ANS-HD and ANS-LD than PLA (*p* < 0.01). $ Significantly higher in AND-LD than PLA. # Significantly higher in ANS-HD than ANS-LD (*p* < 0.01). Anserine effects on CAT levels were not significant (NS).

**Figure 3:**
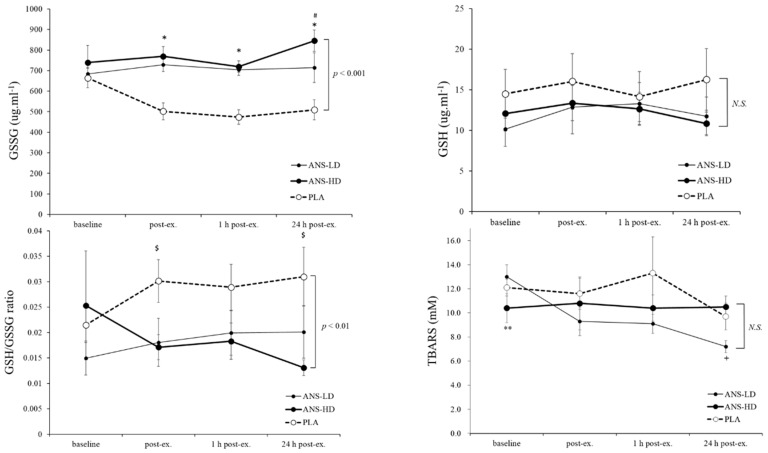
Glutathione disulfide (GSSG), glutathione (GSH), GSH/GSSG ratio, and thiobarbituric acid active reactive substances (TBARS) responses at baseline, immediately post exercise (post-ex), 1 h post-ex, and 24 h post-ex with anserine low dose (ANS-LD), high dose (ANS-HD), and placebo (PLA) treatments. Values are mean ± SEM. GSSG was significantly increased by both ANS-HD and ANS-LD (*p* < 0.001, ANOVA main treatment effect). Main treatment effects of anserine on GSH and TBARS and interaction were not significant (NS). The GSH/GSSG ratio was lower in ANS-HD compared with PLA (*p* < 0.01, ANOVA main treatment effect). * Significantly higher in ANS-HD and ANS-LD than PLA (*p* < 0.01). $ Significantly higher in PLA than ANS-HD and ANS-LD. # Significantly higher in AND-HD than PLA (*p* < 0.05). ** Significantly lower in ANS-HD than PLA (*p* = 0.04). + Significantly lower in ANS-LD than ANS-HD (*p* < 0.05).

Anserine significantly increased SOD (*p* < 0.001, main treatment effects), with no interaction effects. Both ANS-HD and ANS-LD induced an overall 50% higher SOD than PLA (*p* < 0.001, effect size d = 0.81 and 0.79, respectively). These effects were irrespective of baseline levels, with no overall interaction effects (Figure 2). The post exercise (1–24 h) SOD time course response interacted with the treatment (*p* = 0.02 for interaction, and *p* = 0.043 for time effect), with an SOD showing a trend towards a continuous increase from 1 to 24 h post exercise (*p* = 0.051) in ANS-HD, compared with no significant change in ANS-LD or PLA treatments (Figure 2). In contrast, CAT and GSH levels were neither affected by anserine, post exercise time-course, nor was there any interaction effects between the anserine treatment and exercise (Figure 2 and Figure 3).

Anserine significantly increased GSSG (*p* < 0.001, main treatment effects), and both doses ANS-LD (*p* < 0.01) and ANS-HD (*p* < 0.001) increased GSSG compared with PLA, irrespectively of baseline or post exercise time-course with no interaction effects between anserine and post exercise time-course (Figure 3). Anserine also affected the GSH/GSSG ratio (*p* < 0.01, main treatment effects), where both ANS-LD (*p* < 0.01) and ANS-HD (*p* < 0.05) significantly reduced the GSH/GSSG ratio irrespective of post exercise time-course and with no interaction effects (Figure 3).

**Figure 4:**
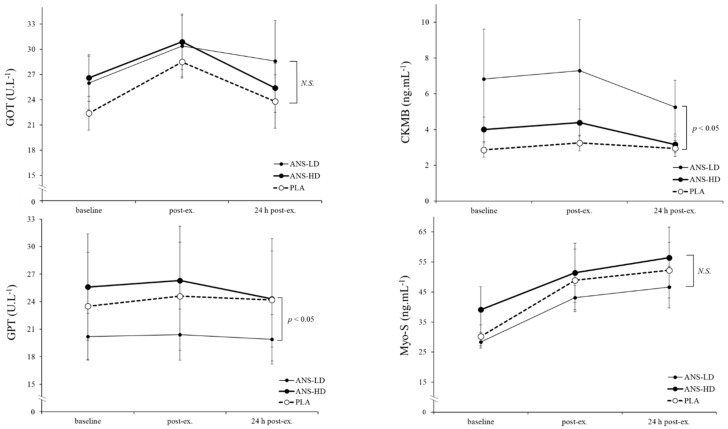
Responses of cell damage biomarkers: Glutamic-oxaloacetic transaminase (GOT), glutamic pyruvic transaminase (GPT), creatine kinase myocardial band (CKMB), and myoglobin (Myo-S) at baseline, immediately post exercise (post-ex) and 24 h post exercise (24 post-ex) with anserine low dose (ANS-LD), high dose (ANS-HD), and placebo (PLA) treatments. Values are mean ± SEM. Higher GPT was found in ANS-HD than ANS-LD (*p* < 0.05), but not PLA. Higher CKMB was found with ANS-HD than PLA (*p* < 0.05), and a trend towards being higher in ANS-LD than PLA (*p* = 0.056). Anserine effects for GOT and Myo-S were not significant (NS).

No overall anserine treatment or interaction effects were found for TBARS (Figure 4), but there was a moderate effect size for reduced TBARS in ANS-LD than PLA (d = 0.43, *p* = 0.17) compared with a low effect size for ANS-HD vs. PLA (d = 0.23, *p* = 0.79). At 24 h post exercise, a lower TBARS was found for ANS-LD than ANS-HD (*p* = 0.02) and a trend towards a lower TBARS in ANS-LD than PLA (*p* = 0.067).

### 3.2. Cell Damage Biomarkers

Anserine significantly affected GPT (*p* < 0.05, main treatment effects) irrespective of post exercise time-course with no interaction effects. ANS-HD induced a higher GPT than ANS-LD (*p* < 0.05) but no difference was found for the GPT response to either anserine dose compared with PLA (Figure 4). Moreover, no GPT interaction effect was found between the anserine treatment and post exercise time-course.

Furthermore, anserine induced significant increases in CKMB (*p* < 0.05, main treatment effects), irrespectively of post exercise time-course, with no interaction effects (Figure 4). ANS-HD induced significantly higher CKMB than PLA (*p* < 0.05), and ANS-LD showed a trend towards being higher in ANS-LD than PLA (*p* = 0.056). GOT and myoglobin levels were neither affected by the anserine dose nor post exercise time-course, nor was there any interaction effects (Figure 4).

### 3.3. Blood Haematology Biomarkers

**Figure 5:**
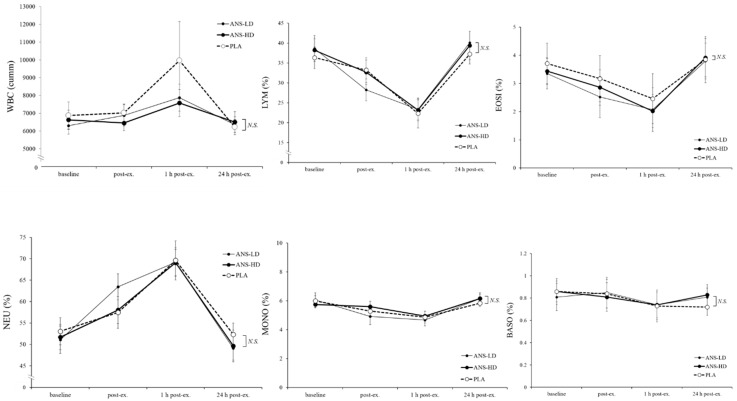
Haematology responses of white blood cells (WBCs) count, neutrophils (NEU), lymphocytes (LYM), monocytes (MONO), eosinophils (ESO), and basophils (BASO) at baseline, immediately post exercise (post-ex), and following 1 and 24 h post-ex with high dose anserine (ANS-HD), low dose anserine (ANS-LD), and placebo (PLA) treatments. Values are mean ± SEM.

Anserine of either dose had no significant effect on any of the haematological WBC biomarkers (neutrophils (NEU); lymphocytes (LYM); monocytes (MONO); eosinophils (EOSI); basophils (BASO)), and no interaction was found between anserine supplementation of either dose with post exercise time-course responses (Figure 5).

**Figure 6:**
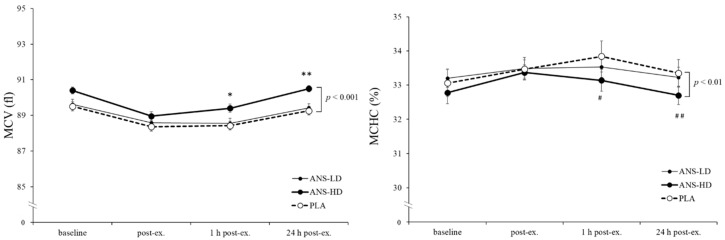
Mean cell volume (MCV) and mean corpuscular haemoglobin concentration (MCHC) responses pre-exercise (baseline), immediately post exercise (post-ex), and throughout recovery: 1 and 24 h with high dose anserine (ANS-HD), low dose anserine (ANS-LD), and placebo (PLA) treatments. Values are mean ± SEM. Significant main treatment effect was found for MCV (*p* < 0.001) and MCHC (*p* < 0.01) following the exercise bout. * Significantly higher ANS-HD than ANS-LD (*p* < 0.05) and PLA (*p* = 0.079). ** Significantly higher ANS-HD than ANS-LD and PLA (*p* < 0.05). # Significantly lower ANS-HD than PLA (*p* < 0.05). ## Significantly lower ANS-HD than PLA and ANS-LD (*p* < 0.05). NS: No significant difference or interaction between the three conditions.

**Figure 7:**
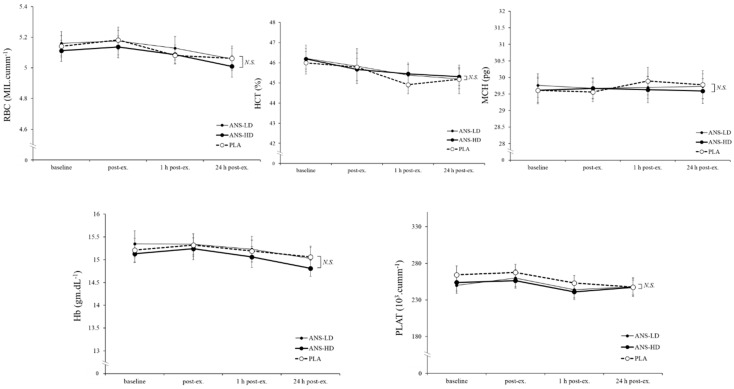
Red blood cells (RBCs), haemoglobin Hb, haematocrit (HCT), mean corpuscular haemoglobin (HCT), and platelet count (PLAT) responses pre-exercise (baseline), immediately post exercise (post-ex), and throughout recovery: 1 and 24 h with high dose anserine (ANS-HD), low dose anserine (ANS-LD), and placebo (PLA) treatments. Values are mean ± SEM. NS: No significant difference or interaction between the three conditions.

RBCs (haemoglobin (Hb); haematocrit (HCT); and mean corpuscular haemoglobin (MCH)) were also unaffected by anserine, its doses, nor was there any interaction effect with post exercise responses (Figure 6). However, compared with ANS-LD and PLA, ANS-HD increased mean cell volume (MCV), (*p* < 0.001 main treatment effects, d = 0.41, 0.35, 0.07 for ANS-HD, ANS-LD, and PLA, respectively), and decreased mean corpuscular haemoglobin concentration (MCHC), (*p* = 0.001, main treatment effects, d = 0.40, 0.35, 0.07 for ANS-HD, ANS-LD, and PLA, respectively) were found, irrespective of post exercise time-course responses, with no interaction effects (Figure 7).

### 3.4. Anserine Effects on Exercise Performance

The TTE exercise performance was not significantly different between the three conditions (2601.9 ± 427.8 s, 2347.9 ± 389.0 s, and 2297.6 ± 309.7 s for PLA, ANS-LD, and ANS-HD, respectively), but Cohen’s effect size d demonstrated a small effect for a higher PLA than ANS-HD (d = 0.26, *p* = 0.17) and ANS-LD (d = 0.22, *p* = 0.37), with no effect size between ANS-LD and ANS-HD (d = 0.05, *p* = 0.85).

## 4. Discussion

This study demonstrates a new role of anserine for antioxidant activity, cell integrity, homeostasis, and adaptive repair following an intense muscular challenge in healthy men. Both ANS-LD and ANS-HD replenished antioxidant levels of SOD and preserved CAT and GSH compared with PLA, without significantly affecting the TBARS levels. However, anserine increased GSSG and consequently reduced the GSH/GSSG ratio, which was concurrent with attenuated cell damage biomarkers of GOT and myoglobin, and a transient increased CKMB and GPT levels. Protective cell homeostasis mechanisms induced by anserine also reflected a haematological increase in RBC volume to concentration and an attenuated WBC elevation.

The present study is the first to demonstrate anserine effects on antioxidant activity, cell integrity, and homeostasis in humans, which confirms previous observations in vitro and in vivo [4,18]. For example, improved cellular homeostasis following short-term anserine injection in oxidative-stressed and glucose co-incubated diabetic mice was explained by an enhanced potent defense mechanism, which ameliorated intracellular oxidative and glycative stress (e.g., increased in heat shock and haemeoxygenase proteins, Hsp70/HO-1) [18]. The antioxidation activity of anserine and carnosine containing a peptide mixture derived from chicken breast has also been explained by enhancing the DPPH (α,α-diphenyl-β-picrylhydrazyl) radical scavenging effect in vitro [4]. In the present human study, anserine replenished the free radical scavenging enzymes SOD and preserved CAT and cofactor GSH suggesting a protection of cellular homeostasis [19]. The 50% increased SOD levels in both ANS-LD and ANS-HD compared with PLA (Figure 2), corresponded to significantly higher AUC for ANS-LD of 41.2% (1.7), and higher AUC for ANS-HD of 37.5% (1.6) compared with AUC for PLA (1.0), *p* < 0.01), (Table 1). Such difference was not present in CAT levels (Figure 2, Table 1).

Protective antioxidant mechanisms of anserine can be attributed to its oxidation reduction effects through modulating genes involved in glucose and lipid metabolism, inflammation, and apoptosis. For example, a hydrolyzed chicken extract supplement (containing anserine) has been shown to regulate Mup-1, -3, -5, -9, -17, -21,-ps16, Ahsg, and Alb genes in male and female mice, which was attributed to increased antioxidant enzymes SOD, CAT, and GSH reductase [10]. Two distinct oxidation reduction pathways have been implicated with the concurrent increase in CAT and SOD levels, one as a consequence of CAT activity through increased GSH ability to scavenge free radicals such as ROS, and another through SOD protection from protein and lipid peroxidation, as well as reduced mitochondrial damage [10]. Therefore, a 50% increased SOD (d = 0.79 and 0.81 for ANS-LD and ANS-HD vs. PLA, respectively) concurrent with unchanged CAT levels (d = 0.1 and 0.2 for ANS-LD and ANS-HD vs. PLA, respectively) suggests a blunted CAT oxidation reduction pathway in which the GSH, GSSG, and GSH/GSSG role were reduced, and consequently TBARS were unchanged (Figure 2 and Figure 3). Conversely, ANS-LD and ANS-HD enhanced the pathway in which SOD protected cellular integrity from peroxidation damage.

Metabolic disturbances and skeletal muscle microdamage induced by a physical exercise challenge in humans are entropic in nature [20,21], and the replenishing effects of novel nutraceuticals on cellular damage and integrity are still being investigated [11,22]. In the present study, cellular damage biomarkers were all within the normal range expected for healthy active participants (~10–40 U/L for GOT and GPT, 0.9–6.1 ng/mL for CKMB, and 18.5–94.8 ng/mL for myoglobin), which is comparable with previously reported data for healthy active or sports participants [13,14,23]. Abnormal change can indicate an acute ischemic trauma or metabolic disease [24]. However, a slight or significant increase in those biomarkers can also be indicative of transient adaptive changes following a strenuous exercise challenge such as the TTE within the present study. Significant increases in GOT and GPT (22 and 12 U/L at baseline to 46 and 23 U/L after exercise) [13,23] and in CKMB and myoglobin [14] have been reported following exhaustive exercise bouts. Transient elevations of GOT and GPT have also been reported following a nutrient intake [25]. Indicators of cell damage often subsides as a result of exercise recovery, adaptation to repeated exercise bouts, or nutritional intake. For example, the CKMB increase from 2 to 9 mg/dl returned to baseline levels following 48 h recovery in amateur male triathletes [14]. Such time-dependent effects on cell damage biomarkers (CKMB, GOT, and GPT) were observed in our data following a shorter recovery time (24 h), but were not significant (Figure 4) possibly due to an elevated baseline level in the ANS-LD and ANS-HD treatments. The present data suggests that transient cell damage biomarker changes are indicative of an initiation of longer-term adaptive response to a challenge such as exercise.

Anserine’s potential for augmenting cell damage repair adaptations may have reflected a preserved WBC (Figure 5). However, RBC levels showed a small but significant increase in MCV (*p* < 0.01) and a decrease (~1%) in MCHC (*p* = 0.001) in ANS-HD compared with ANS-LD and PLA (Figure 6), which is close to previously reported decreased MCHC following either intense exercise training (~1%–3%) [15] or a high intensity exercise training combined with a vitamin D supplement (~0.7%) [26]. Senescent RBCs are particularly prone to exercise-induced intravascular haemolysis and an associated decrease in density and density distribution compared with younger less dense RBCs [27]. Nonetheless, future long-term adaptations of exercise and anserine supplementation effects on haematological and associated cell damage biomarkers warrant further studies.

Similar ANS-LD and ANS-HD benefits on antioxidative and cell protection mechanisms suggest that ANS-LD (~15 mg·kg^−1^·bw^−1^) is nutritionally adequate, which is in line with the recently recommended range of anserine doses of 15–20 mg·kg^−1^·bw^−1^ used to detect its bioavailability and time-course synthesis [9]. Anserine offers a new sports recovery supplement following an exhaustive performance of TTE, as shown by various post exercise effects on cell homeostasis biomarkers measured within this study. However, lack of the TTE significant differences between ANS-LD, ANS-HD, and PLA rules out anserine’s acute ergogenic enhancement of the TTE endurance performance, though testing different exercise protocols with various intensities and durations may elicit different outcomes.

In terms of our study’s limitations, the participants reported maintaining a similar dietary intake, but an analysis of diet diaries would be more accurate in ensuring dietary compliance. Another limitation is the single TTE session used in the present study and other studies with a similar design [22], which may have only triggered a slight response in some of the oxidative stress and cell damage biomarkers [28]. Our study’s exercise protocol allowed sufficient duration (~40 min) for detecting the biosynthesis effects of anserine in healthy active participants. We used a two-week wash-out period between the TTE bouts, which is in line with similarly designed supplementation studies [29]. Such wash-out period, though practical in healthy active participants of this study, cannot rule out potential protective muscle damage effects (e.g., lowered CKMB following a second intense exercise bout), which can be mitigated by longer recovery periods (e.g., more than four weeks) [30], but such debate is beyond the scope of this study. The TTE intensity estimated based on 50%$\dot{V}$O_2peak_ may be considered low for triggering some antioxidative and muscle damage biomarkers (e.g., CKMB, myoglobin), especially those requiring a longer recovery period (48 h) following more intense forms of exercise [14]. Our estimates of the TTE speed and gradient (average velocity of 6.3 km·h^−1^ and gradient of 12.8%, which corresponded to 71.6% ± 2.5% and 64.6% ± 1.2% of the participant’s maximum speed and gradient, respectively) from the Bruce protocol may have underestimated the actual TTE %$\dot{V}$O_2peak_ intensity, which suggests that participants may have exercised at a slightly higher intensity than 50%$\dot{V}$O_2peak_, given the responses found in the biomarkers tested. Future protocols could look at such responses following higher exercise intensities using more direct measurements of TTE %$\dot{V}$O_2peak_ intensity, or with repeated bouts and following longitudinal adaptations to interventions combining exercise training with an anserine supplement, especially in patients with elevated oxidative stress markers. Nonetheless, our study is the first to demonstrate the combined oxidative stress, cell damage integrity, and homeostasis responses induced by exercise in humans.

## 5. Conclusions

Anserine supplementation improves antioxidant activity and cellular protection by alleviating exercise-induced oxidative stress and preserving cellular homoeostasis in healthy men. However, anserine’s temporal elevation of exercise-induced cell damage, together with enhanced antioxidative properties and haematology changes suggest an initiation of long-term exercise-induced adaptations. Anserine cell repair and antioxidation effects promote it as an effective nutraceutical for chronic disease prevention and recovery from intense exercise.

## Figures and Tables

**Table 1:** Area under the curve with high dose anserine (ANS-HD), low dose anserine (ANS-LD), and placebo (PLA) treatments of the following blood biomarkers: Oxidative stress; superoxide dismutase (SOD), catalase (CAT), glutathione disulfide (GSSG), glutathione (GSH), GSH/GSSG ratio, and thiobarbituric acid active reactive substances (TBARS); cell damage; glutamic-oxaloacetic transaminase (GOT), glutamic pyruvic transaminase (GPT), creatine kinase myocardial band (CKMB), and myoglobin (Myo-S); haematology red blood cells; mean cell volume (MCV), mean corpuscular haemoglobin concentration (MCHC), red blood cells (RBCs), haemoglobin (Hb), haematocrit (HCT), mean corpuscular haemoglobin (HCT), and platelet count (PLAT); haematology white blood cells (WBCs) count, neutrophils (NEU), lymphocytes (LYM), monocytes (MONO), eosinophils (ESO), basophils (BASO). Values are mean ± SEM.

Blood Biomarkers	Area Under the Curve (AUC) (mean ± SEM)			*p*-Values (Main ANOVA Effect)	*p*-Values (Post Hoc)		
	PLA	ANS-LD	ANS-HD		PLA vs. ANS-LD	PLA vs. ANS-HD	ANS-LD vs. ANS-HD
CAT (nmol·min^−1^·mL^−1^)	900.8 ± 80.3	919.5 ± 67.4	994.7 ± 87.6	0.21	0.43	0.22	0.14
SOD (U/mL)	1.0 ± 0.1	1.7 ± 0.1	1.6 ± 0.1	0.12	<0.01	<0.01	0.31
GSH (ug/mL)	91.2 ± 19.2	74.2 ± 11.4	74.9 ± 8.8	<0.05	0.17	0.14	0.46
GSSG (ug/mL)	3123.8 ± 213.6	4262.1 ± 122.1	4561.2 ± 175.7	0.61	<0.01	<0.01	0.08
GSH/GSSG Ratio	0.2 ± 0.0	0.1 ± 0.0	0.1 ± 0.0	0.86	<0.01	<0.01	0.45
TBARS (μM)	71.6 ± 7.9	57.2 ± 3.7	63.3 ± 5.6	0.08	0.055	0.14	0.07
GOT (U/L)	103.2 ± 8.7	115.4 ± 14.5	113.8 ± 12.1	0.05	0.20	0.06	0.45
GPT (U/L)	96.9 ± 24.3	80.9 ± 10.7	102.5 ± 22.8	<0.05	0.15	0.15	0.06
CKMB (ng/mL)	12.3 ± 1.6	12.7 ± 2.4	16.0 ± 2.5	0.08	0.38	0.057	0.12
Myo-S (ng/mL)	232.5 ± 38.1	227.6 ± 23.1	254.8 ± 46.3	0.39	0.42	0.26	0.25
WBC (cumm)	47075 ± 5060.2	42177 ± 2863.6	41210 ± 2478.7	0.08	0.09	0.11	0.33
LYM (%)	184.7 ± 16.1	181.9 ± 14.2	189.4 ± 15.3	0.07	0.32	0.35	0.22
EOSI (%)	18.8 ± 4.9	16.3 ± 4.1	17.1 ± 3.4	0.93	0.14	0.21	0.35
NEU (%)	359.6 ± 19.8	365.6 ± 14.7	355.6 ± 16.6	0.21	0.23	0.38	0.18
MONO (%)	32.2 ± 2.0	31.4 ± 2.5	33.0 ± 1.7	<0.01	0.36	0.14	0.19
BASO (%)	4.7 ± 0.7	4.8 ± 0.7	4.8 ± 0.6	0.85	0.36	0.37	0.48
MCV (fl)	532.3 ± 4.2	533.3 ± 4.0	537.6 ± 4.3	0.17	0.31	<0.05	<0.01
MCHC (%)	201.0 ± 2.1	200.4 ± 1.5	198.5 ± 1.4	0.12	0.29	<0.01	<0.01
RBC (MIL/cumm)	30.7 ± 0.3	30.8 ± 0.5	30.6 ± 0.4	0.65	0.35	0.21	0.16
HCT (%)	272.6 ± 3.1	273.9 ± 3.9	273.7 ± 2.7	0.60	0.30	0.26	0.47
MCH (pg)	178.3 ± 2.2	178.2 ± 1.9	177.8 ± 2.0	0.71	0.45	0.14	0.15
Hb (g/dL)	91.3 ± 1.4	91.5 ± 1.5	90.5 ± 1.3	0.39	0.38	0.08	0.09
PLAT (10^3^/cumm)	1553.3 ± 62.0	1505.4 ± 67.3	1496.3 ± 62.2	0.35	0.24	0.12	0.42
